# Efficacy and safety of fecal microbiota transplantation in the treatment of ulcerative colitis: a systematic review and meta-analysis

**DOI:** 10.1038/s41598-023-41182-6

**Published:** 2023-09-03

**Authors:** Jing Feng, Yexin Chen, Yan Liu, Lin Lin, Xiujuan Lin, Wenxiu Gong, Rongmu Xia, Jianquan He, Jianwen Sheng, Huimei Cai, Chuanxing Xiao

**Affiliations:** 1 Department of Gastroenterology, Shanxi Provincial People’s Hospital, The Fifth Hospital of Shanxi Medical University, Taiyuan, 030012 China; 2 School of Pharmacy, Fujian University of Traditional Chinese Medicine, Fuzhou, 350122 China; 3 Xiamen Treatgut Biotechnology Co., Ltd, Xiamen, 361101 China; 4 Department of Gastroenterology, Chongqing General Hospital, Chongqing, 401147 China; 5 Department of Gastroenterology, The Second Affiliated Hospital of Fujian University of Traditional Chinese Medicine, No.282, Wusi Road, Fuzhou, 350003 China; 6 College of Rehabilitation Medicine, Fujian University of Traditional Chinese Medicine, Fuzhou, 350122 China; 7 Department of Gastroenterology, Yichun People’s Hospital, The Affiliated Hospital of Yichun University, No 1061, Jinxiu Road, Yichun, 336000 China; 8 Department of Gastroenterology, Fuzhou First Hospital Affiliated to Fujian Medical University, No. 190, Dadao Road, Fuzhou, 350009 China

**Keywords:** Microbiology, Gastroenterology

## Abstract

To explore the efficacy and safety of fecal microbiota transplantation (FMT) as a treatment approach for ulcerative colitis (UC), a comprehensive systematic review and meta-analysis of randomized controlled trials was conducted. To collect and evaluate randomized controlled trials of high quality on FMT for UC, we searched a number of databases, including PubMed, Web of Science, Cochrane, Embase, and Medline, for studies published between the establishment of the databases and March 2023. We conducted a meta-analysis of the studies using Review Manager software (version 5.4.1) to determine the differences in rates of remission and adverse reactions between the FMT group and the control group, utilizing the risk ratio (*RR*) and 95% confidence interval (*CI*) to combine our findings. A total of 13 randomized controlled trials (RCTs) on the efficacy of FMT in patients with UC were included in the study, in which 580 patients participated, including 293 patients treated with FMT and 287 control subjects. Meta-analysis revealed that clinical remission was significantly better in the FMT group than in the control group [*RR* = 1.73; 95% *CI* = (1.41, 2.12); *P* < 0.00001]; endoscopic remission was significantly better in the FMT group than in the control group [*RR* = 1.74; 95% *CI* = (1.24, 2.44); *P* = 0.001]. Additionally, there were no significant differences in the incidence of adverse reactions between the two groups [*RR* = 1.00; 95% *CI* = (0.86, 1.15); *P* = 0.96]. Fecal microbiota transplantation has shown potential as a therapeutic intervention for inducing clinical remission in ulcerative colitis UC; nevertheless, the attainment of endoscopic remission and the maintenance of long-term remission continue to present challenges. Safety concerns persist throughout the treatment process, necessitating the implementation of measures to augment both safety and success rates.

## Introduction

Ulcerative colitis (UC) is a chronic, nonspecific inflammatory disease of the colon and rectum that falls under the category of inflammatory bowel diseases (IBD). Its clinical symptoms include diarrhea, hematochezia, and abdominal pain. The pathogenesis of UC is closely related to defects in colonic epithelial cells, mucus barrier, and epithelial barrier^1^. The development of UC is influenced by several key factors, including genetics, immune system dysregulation, intestinal microbiota dysbiosis, and environmental factors. Unfortunately, UC's incidence is increasing globally, and it is a highly debilitating condition for many patients.

UC can be treated with various pharmacological approaches, such as aminosalicylates, glucocorticoids, biological agents, and immunosuppressants. However, despite the wide range of options available, there are still cases where patients remain unresponsive to treatment, or the therapeutic effects are not significant enough^2^. This underscores the need for the formulation and implementation of novel therapeutic strategies to address these challenges.

The intestine plays a crucial role as the largest immune organ in the human body, contributing up to 70% of the immune function. Additionally, it represents the largest microecosystem in the host. The human intestinal microbiota is primarily comprised of four phyla: *Firmicutes, Bacteroidetes*, *Proteobacteria,* and *Actinobacteria*^3^. If the intestinal microbiota becomes dysregulated, it can lead to a decline in the defensive and immunomodulatory functions of the gut. This, in turn, increases the risk of developing various diseases due to the associated pathogenic factors^4^. Gut microbiota dysbiosis is a vital factor in the development of UC^5^, which usually includes abnormal distribution of gut microbiota and reduced biodiversity and abundance of intestinal commensal microorganisms. Gut dysbiosis in UC patients manifests as a decrease in the proportion of Firmicutes and an increase in the proportion of Proteobacteria^3, 6, 7^.

A novel therapy for UC that aims to improve the diversity and abundance of the gut microbiota has been developed: fecal microbiota transplantation (FMT)^8, 9^. This involves transplanting fecal bacteria from a donor to a recipient, which has been shown to enhance the abundance of gut microbiota in UC patients and restore their function. FMT's potential for clinical therapeutic value in UC is significant.

Several countries have conducted research to examine the effectiveness and safety of FMT as a treatment for UC^8–10^. However, there is inconsistency in the methods utilized across these studies, leading to varying results. As such, there is a need to further consider both the efficacy and safety of FMT for the treatment of UC. To gain a deeper understanding of the effectiveness and safety of FMT in treating UC, a systematic review and meta-analysis has been conducted using high-quality data from randomized controlled trials (RCTs). The objective is to evaluate and update our knowledge on the efficacy and safety of FMT in treating UC, providing valuable insights and evidence to guide clinicians and healthcare professionals in their decision-making regarding the use of FMT in the management of ulcerative colitis patients.

## Methods

### Search strategy

To gather comprehensive data, we conducted searches in various databases including PubMed, Web of Science, Cochrane, Embase, and Medline, covering the time span from the inception of these databases up until March 2023. Our literature searches were executed using a specific set of key terms: ((FMT or fecal microbiota transplantation or intestinal microbiota transplantation or bacteriotherapy) OR (feces or stool) AND (transplant or implant or instillation or infusion or transfer or reconstitution or enema or colonoscopy or nasogastric tube or donor)) AND (UC or ulcerative colitis). The scope of this study was limited to original research that was published in the English language. Two authors were responsible for conducting the literature search and assessing the results, and any discrepancies were brought to the attention of the senior researcher for resolution through further discussion.

### Inclusion and exclusion criteria

To select literature for this study, we employed a set of inclusion criteria based on the principles of evidence-based medicine (PICOS). The criteria were as follows: (I) the study had to be a randomized controlled trial; (II) the participants had to be patients with ulcerative colitis; (III) the intervention had to be FMT, which could be administered orally, via colonoscopy, nasogastric tube, or enema. Any study that met these criteria was included in the analysis. In this study, the control measure utilized either a placebo or an appropriate control treatment to ensure accurate results. The outcome indicators measured the efficacy of FMT treatment, as well as the safety of the intervention. The exclusion criteria for this study are as follows: reviews, conference papers, studies involving animals, in vitro trials, case–control studies, case series reports, and cohort studies have been excluded from the scope. Additionally, any articles that contain repeated publications or incomplete data have also been excluded.

### Quality assessment

The Cochrane risk of bias tool was employed to evaluate the risk of bias in all of the studies that were included in this paper. The tool evaluates the following seven areas: (I) random sequence generation (selection bias), (II) allocation concealment (selection bias), (III) blinding of participants and personnel (performance bias), (IV) blinding of outcome assessment (detection bias), (V) incomplete outcome data (attrition bias), (VI) selective reporting (reporting bias), and (VII) other bias. The level of bias risk was evaluated and categorized as either "low risk," "high risk," or "unclear risk."

### Data extraction

Two authors worked independently to identify and compile the essential data from the article. They then cross-checked the collected results to ensure consistency. The extracted information comprised details such as the author’s name, publication year, country, patient type, FMT mode, control mode, delivery route, donor type, evaluation time, total FMT dose, number of clinical remissions, number of endoscopic remissions, and number of adverse reactions. The extracted data was then organized into tables for easy reference.

### Statistical analysis

In this study, we utilized Review Manager software (version 5.4.1) to conduct a meta-analysis comparing remission and adverse reaction rates between the FMT and control groups by combining the risk ratio (*RR*) and 95% confidence interval (*CI*). Heterogeneity was assessed using the *Chi*^*2*^ test, and the *I*^*2*^ index was used as an index of heterogeneity. When the *I*^*2*^ value was < 50%, the heterogeneity among the studies was low and the fixed effect model (FEM) was used for the meta-analysis; otherwise, the heterogeneity among the studies was considered high and the random effect model (REM) was used. In addition, we conducted subgroup analyses based on various factors such as delivery route, donor type, evaluation time, total dose of FMT, control mode, and literature publication time. All statistical tests were two-tailed and a significance level of *P* < 0.05 was established.

## Results

### Search results

**Figure 1:**
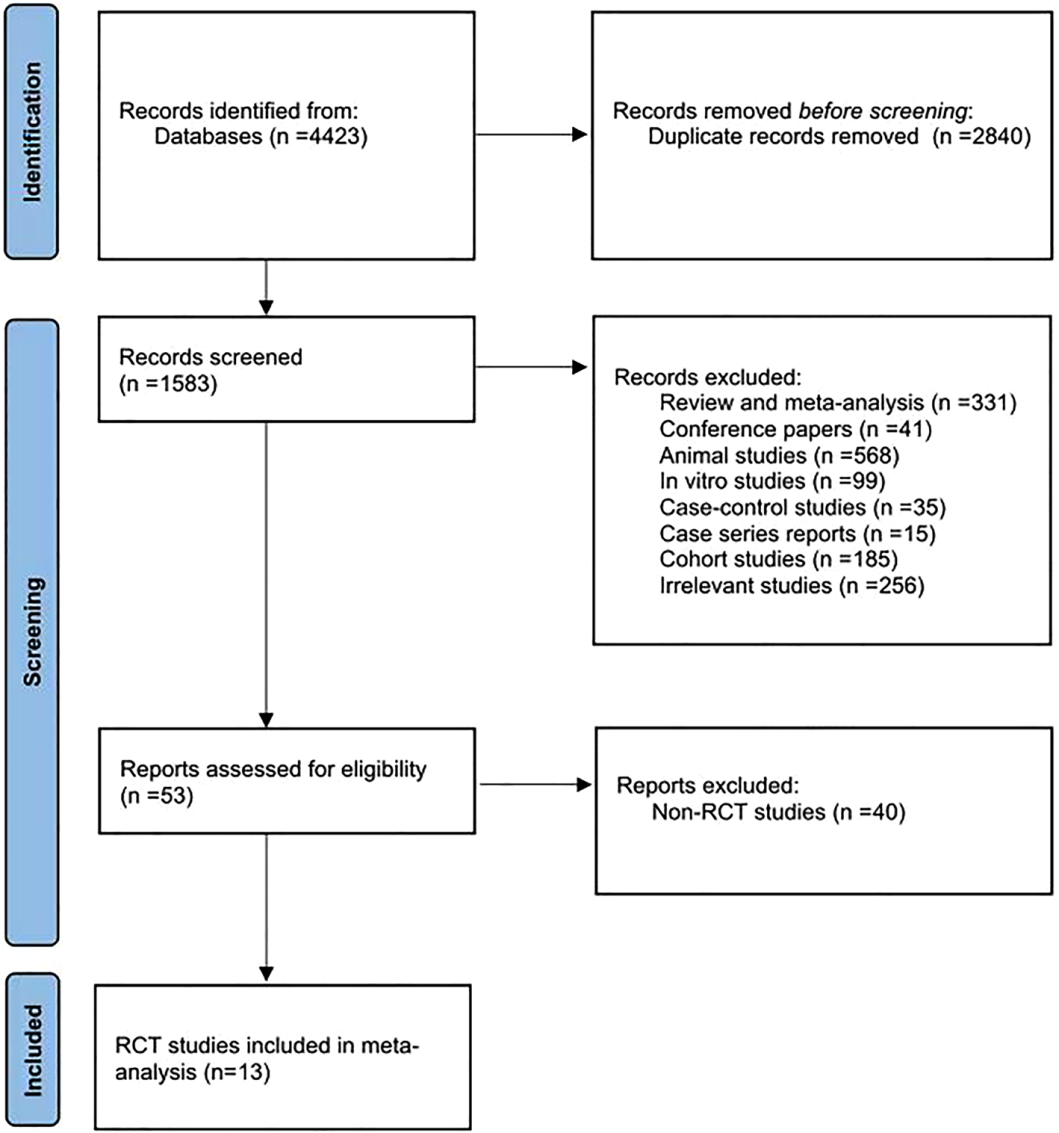
Flow diagram of literature screening.

Through a literature search, a total of 4423 articles were identified. After duplicates were removed, 1583 articles underwent evaluation, with the decision to include based on the titles and their respective abstracts. Following independent screening of titles and abstracts by two authors, 53 clinical studies on the treatment of patients with UC by FMT were ultimately retained. Thirteen RCTs investigating the effectiveness of FMT in patients with UC were identified from the literature, and these studies were included in the meta-analysis^8–20^. We created a PRISMA flow diagram following the PRISMA 2020 guidelines. The literature screening process is illustrated in Fig. 1. These exclusions encompassed various types of studies, including review and meta-analysis (N = 331), conference papers (N = 41), animal studies (N = 568), in vitro studies (N = 99), case–control studies (N = 35), case series reports (N = 15), cohort studies (N = 185), and irrelevant studies (N = 256). The studies were published between 2015 and 2022, and the total number of participants was 580, comprising 293 patients who received FMT and 287 control patients. Relevant details from the studies are presented in Tables 1 and 2 for ease of reference.

**Table 1:** Characteristics of the studies.

Study	Year	Country	Patient type	FMT mode	Control mode	Delivery route	Donor type	Evaluation time	Total dose of FMT
Kedia et al.^8^	2022	India	Mild to moderate UC	FMT-AID	SMT	Colonoscopy	Multiple donors	Week 8	350.0 g
Sarbagili et al.^9^	2022	Israel	Active UC	FMT + UCED	UCED	Colonoscopy and enema	Single donor	Week 8	133.3 g
Haifer et al.^10^	2022	Australia	Active UC	FMT	Placebo	Oral capsules	Single donor	Week 8	102.9 g
Crothers et al.^11^	2021	United States	Mild to moderate UC	FMT	Placebo	Colonoscopy and oral capsules	Multiple donors	Week 12	90.0 g
Březina et al.^12^	2021	Czech Republic	Active left-sided UC	FMT	5-ASA	Enema	Single donor	Week 12	500.0 g
Pai et al.^13^	2021	Canada	Pediatric active UC	FMT	Placebo	Enema	Multiple donors	Week 30	600.0 g
Fang et al.^14^	2021	China	Recurrent active UC	FMT	5-ASA	Colonoscopy	Single donor	Week 8	50.0 g
Schierová et al.^15^	2020	Czech Republic	Active left-sided UC	FMT	5-ASA	Enema	Single donor	Week 12	500.0 g
Sood et al.^16^	2019	India	Active UC	FMT + SOC	Placebo + SOC	Colonoscopy	Single donor	Week 48	700.0 g
Costello et al.^17^	2019	Australia	Mild to moderate active UC	FMT	Autologous FMT	Colonoscopy and enema	Multiple donors	Week 8	100.0 g
Paramsothy et al.^18^	2017	Australia	Active UC	FMT	Placebo	Colonoscopy and enema	Multiple donors	Week 8	1537.5 g
Rossen et al.^19^	2015	The Netherlands	Mild to moderate active UC	FMT	Autologous FMT	Nasoduodenal tube	Single donor	Week 12	240.0 g
Moayyedi et al.^20^	2015	Canada	Active UC	FMT	Placebo	Enema	Single donor	Week 7	300.0 g

FMT-AID, faecal microbiota transplantation with anti-inflammatory diet; SMT, standard medical therapy; UCED, ulcerative colitis exclusion diet; SOC, standard of care therapy.

**Table 2:** Efficacy and safety data of the studies.

Study	Year	FMT(clinical remission)		Control(clinical remission)		FMT(endoscopic remission)		Control(endoscopic remission)		FMT(adverse reactions)		Control(adverse reactions)	
		Events	Total	Events	Total	Events	Total	Events	Total	Events	Total	Events	Total
Kedia et al.^8^	2022	21	35	10	31	12	33	4	23	26	35	27	31
Sarbagili et al.^9^	2022	4	19	6	15	3	19	4	15	NR	NR	NR	NR
Haifer et al.^10^	2022	11	15	5	20	7	15	3	20	10	15	17	20
Crothers et al.^11^	2021	2	6	0	6	NR	NR	NR	NR	2	6	2	6
Březina et al.^12^	2021	12	21	8	22	3	21	3	22	12	21	13	22
Pai et al.^13^	2021	5	12	4	12	NR	NR	NR	NR	5	12	1	12
Fang et al.^14^	2021	9	10	5	10	NR	NR	NR	NR	0	10	0	10
Schierová et al.^15^	2020	3	8	4	8	1	8	3	8	0	8	0	8
Sood et al.^16^	2019	27	31	20	30	18	31	8	30	0	31	0	30
Costello et al.^17^	2019	18	38	6	35	4	38	0	35	3	38	2	35
Paramsothy et al.^18^	2017	18	41	8	40	5	41	3	40	32	41	33	40
Rossen et al.^19^	2015	7	17	5	20	8	17	9	20	18	23	16	25
Moayyedi et al.^20^	2015	9	38	2	37	9	38	2	37	3	38	2	37

NR, not reported.

The studies analyzed in this subject paper primarily focused on mild to moderate UC patients with a Mayo score of 4–10 and an endoscopic Mayo subscore of ≥ 1. The control modes in these studies consisted of placebo, autologous FMT, 5-ASA, and standard drug therapy, while the transplantation methods used included colonoscopy, enema, oral capsule, and nasoduodenal tube transplantation. Regarding donor selection, there were both single donor and multiple donors utilized in these studies. The text displays a range of multiple donors, with a count that fluctuates between 2 and 7. In most of the studies included, clinical remission was the primary outcome, with endoscopic remission serving as the secondary outcome. The clinical remission was defined as a Mayo score of ≤ 2, with each Mayo subscore being no more than ≤ 1. On the other hand, endoscopic remission was defined by an endoscopic Mayo score of ≤ 1. The studies were evaluated between week 8 and week 48, with a greater focus on weeks 8 and 12. When multiple evaluation times occurred in one study, we selected data that shared similar evaluation periods to other studies to maintain consistency as much as possible.

### Quality assessment

**Figure 2:**
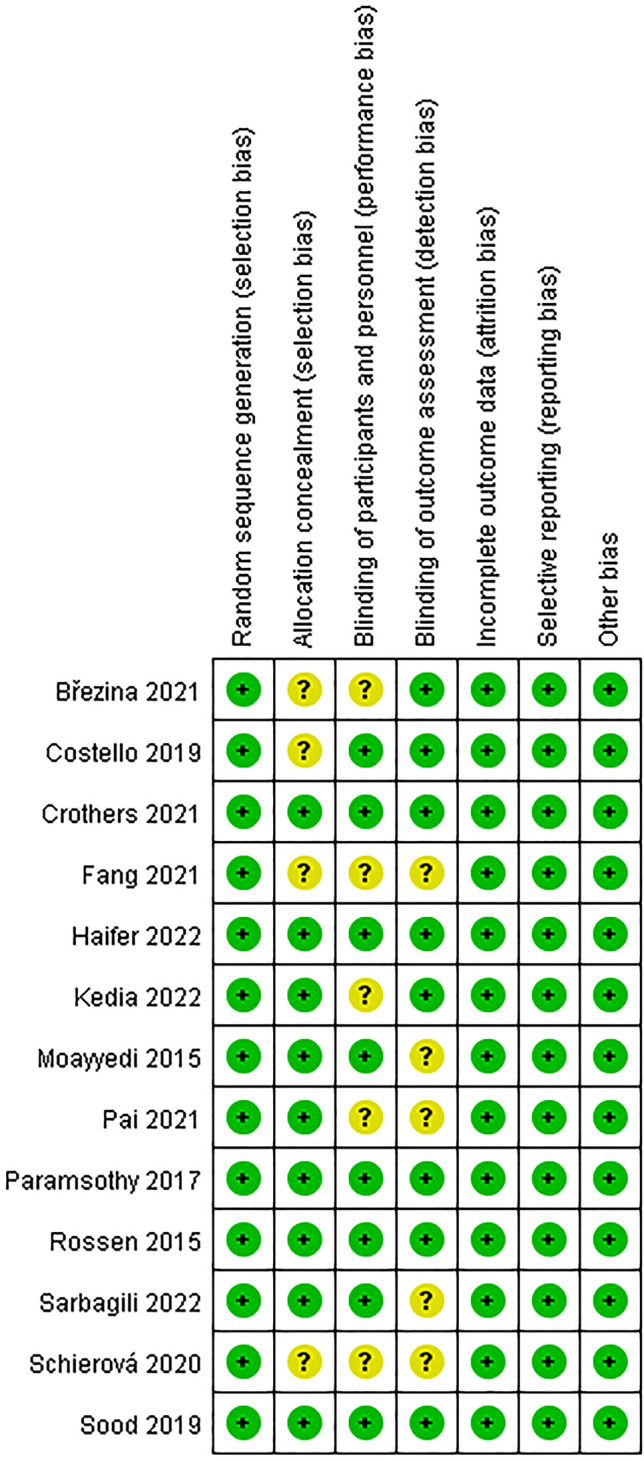
Quality assessment of the studies.

**Figure 3:**
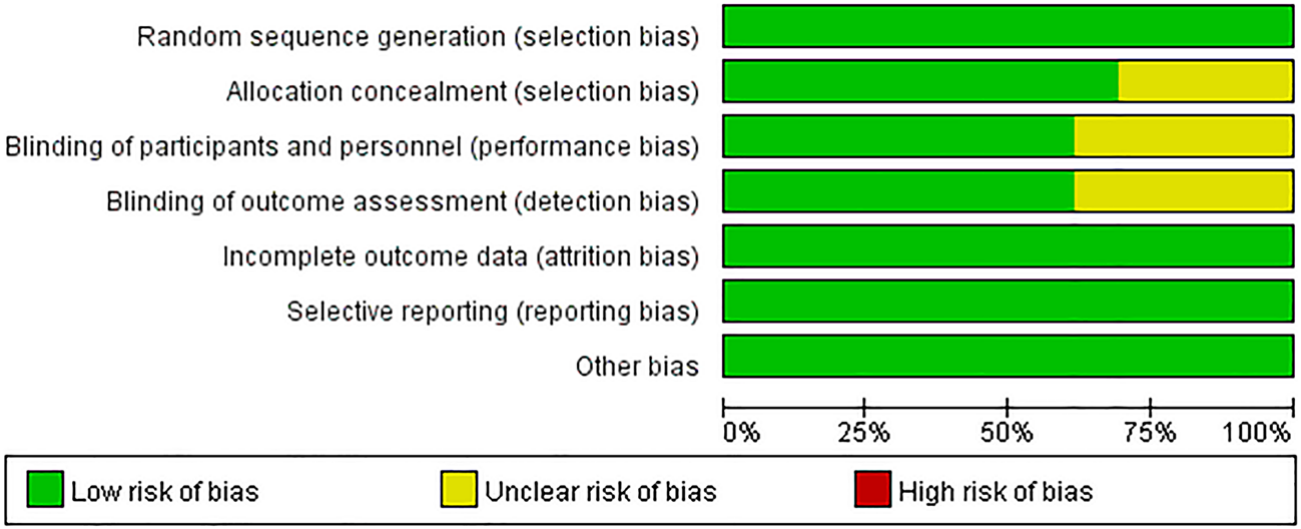
Percentage of risk of bias in the studies.

To evaluate the quality of the studies included, we utilized the Cochrane risk of bias tool (Fig. 2 and Fig. 3). Through this process, we found a small number of studies in which the allocation concealment was not clearly stated and a small number of studies in which blinding was incompletely implemented, and they were assessed as having an unclear risk of bias. Nonetheless, all of the studies involved underwent randomization and were deemed to have a low risk of bias related to elements such as attrition bias and reporting bias, as well as other potential sources of bias.

### Meta-analysis of clinical remission

**Figure 4:**
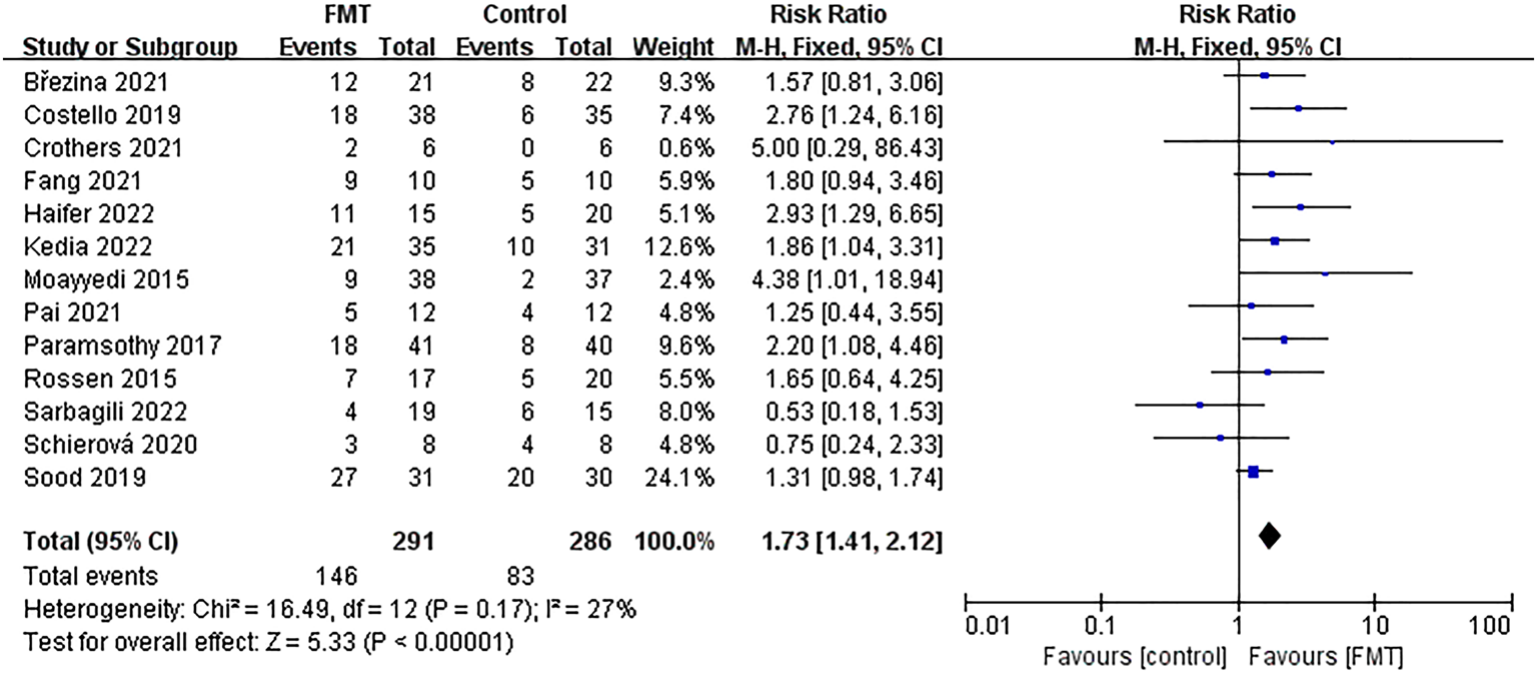
Forest plot for meta-analysis of clinical remission.

All of the studies included in this analysis reported data on clinical remission outcomes. Clinical remission was achieved in 146 out of 291 patients (50.17%) in the group that received FMT and 83 out of 286 patients (29.02%) in the control group. These results suggest that the FMT group had significantly better clinical remission than the control group [*RR* = 1.73; 95% *CI* = (1.41, 2.12); *P* < 0.00001]. Furthermore, our meta-analysis found low levels of heterogeneity between studies (*Chi*^*2*^ = 16.49; *P* = 0.17; *I*^*2*^ = 27%) (Fig. 4).

Next, a funnel plot was utilized to analyze publication bias in the included studies (Supplementary Fig. 1). Data analysis indicated a symmetric distribution. To further investigate potential publication bias, Begg's test was performed and had a *P*-value of 0.9514, while Egger's test had a *P*-value of 0.2334, indicating no observed publication bias.

### Subgroup analysis of clinical remission

To further assess how various study methods influence the effectiveness of FMT, we conducted subgroup analyses on clinical remission data. These analyses were focused on multiple factors, including the delivery route, donor type, evaluation time, total FMT dosage, control mode, and publication time of related literature.

**Figure 5:**
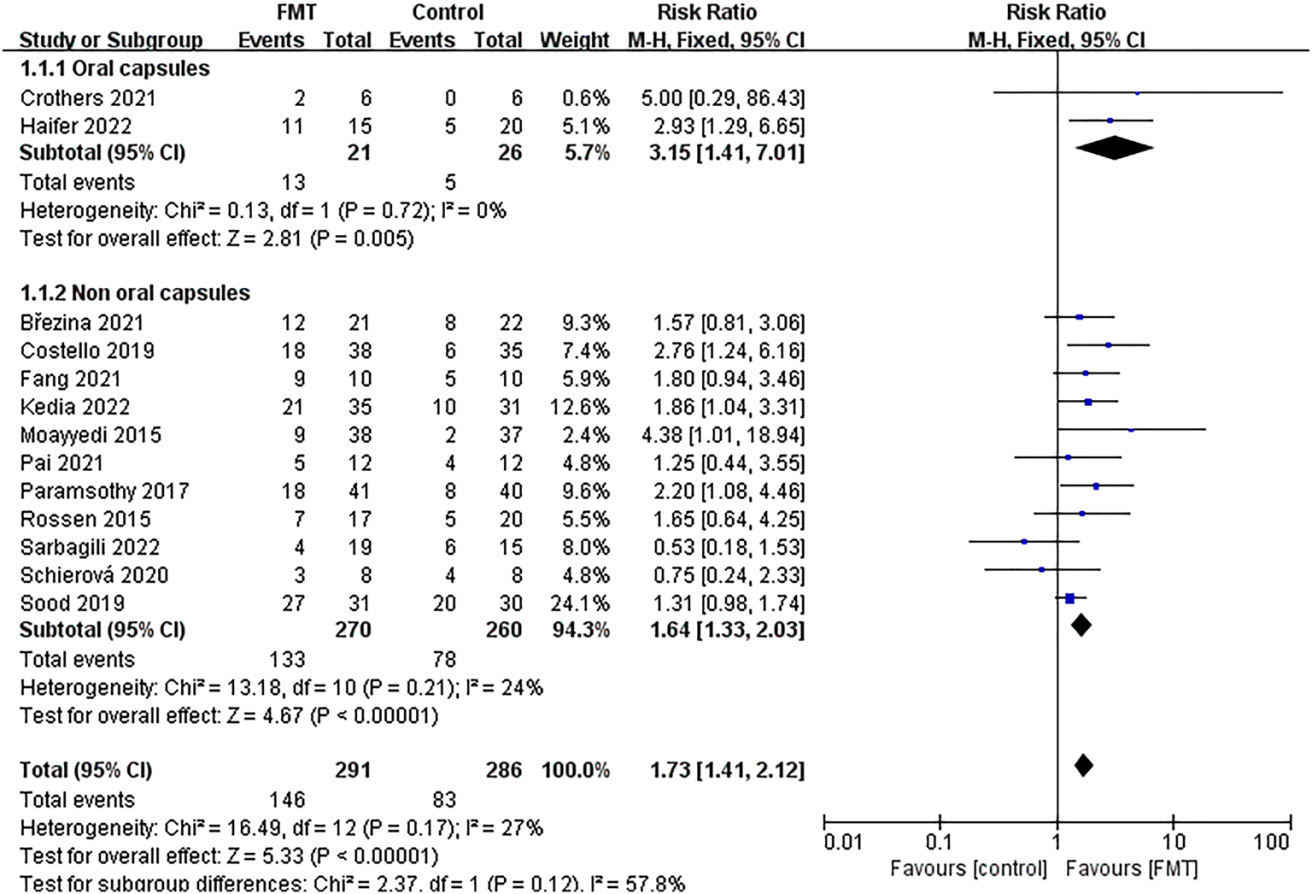
Subgroup analysis of clinical remission of delivery route.

**Figure 6:**
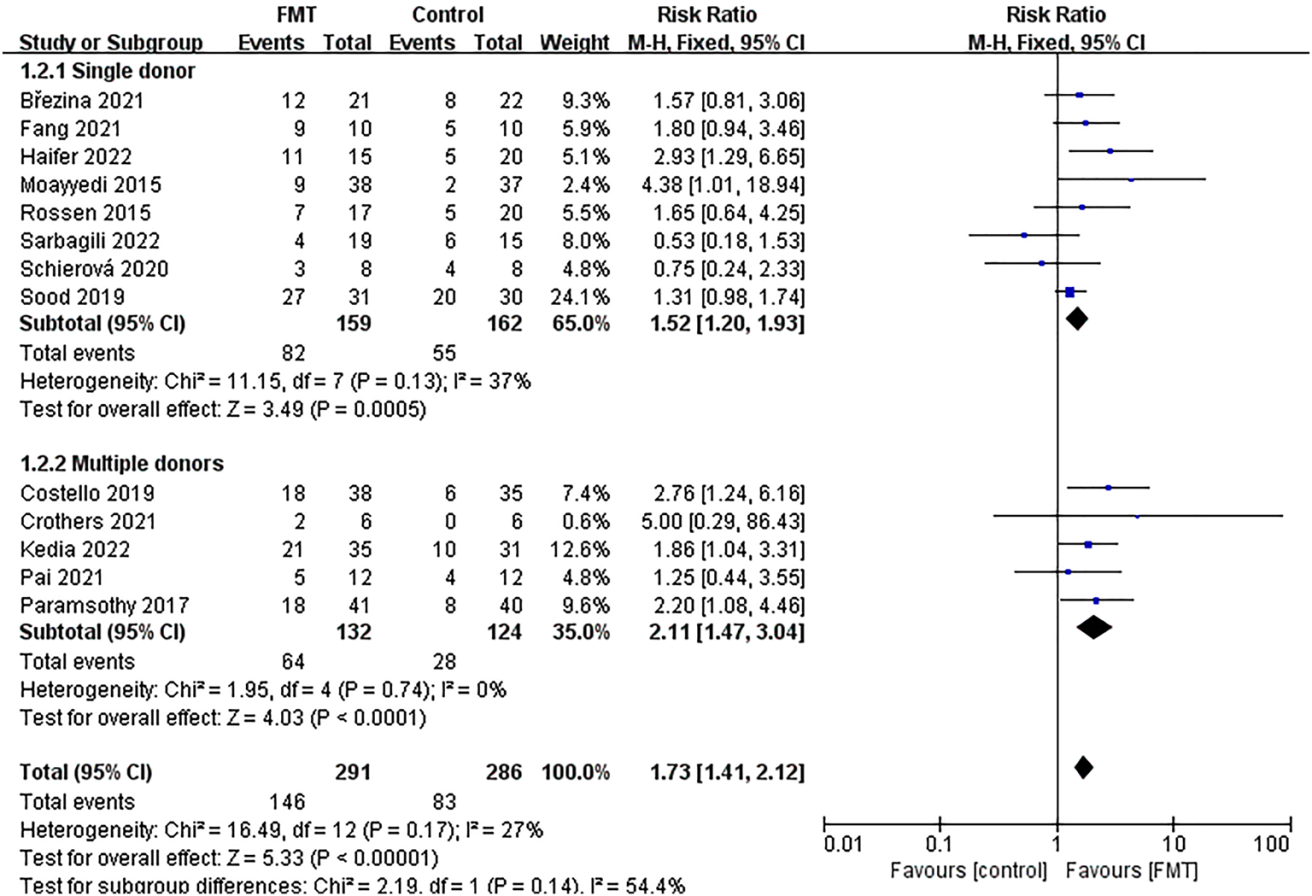
Subgroup analysis of clinical remission of donor type.

**Figure 7:**
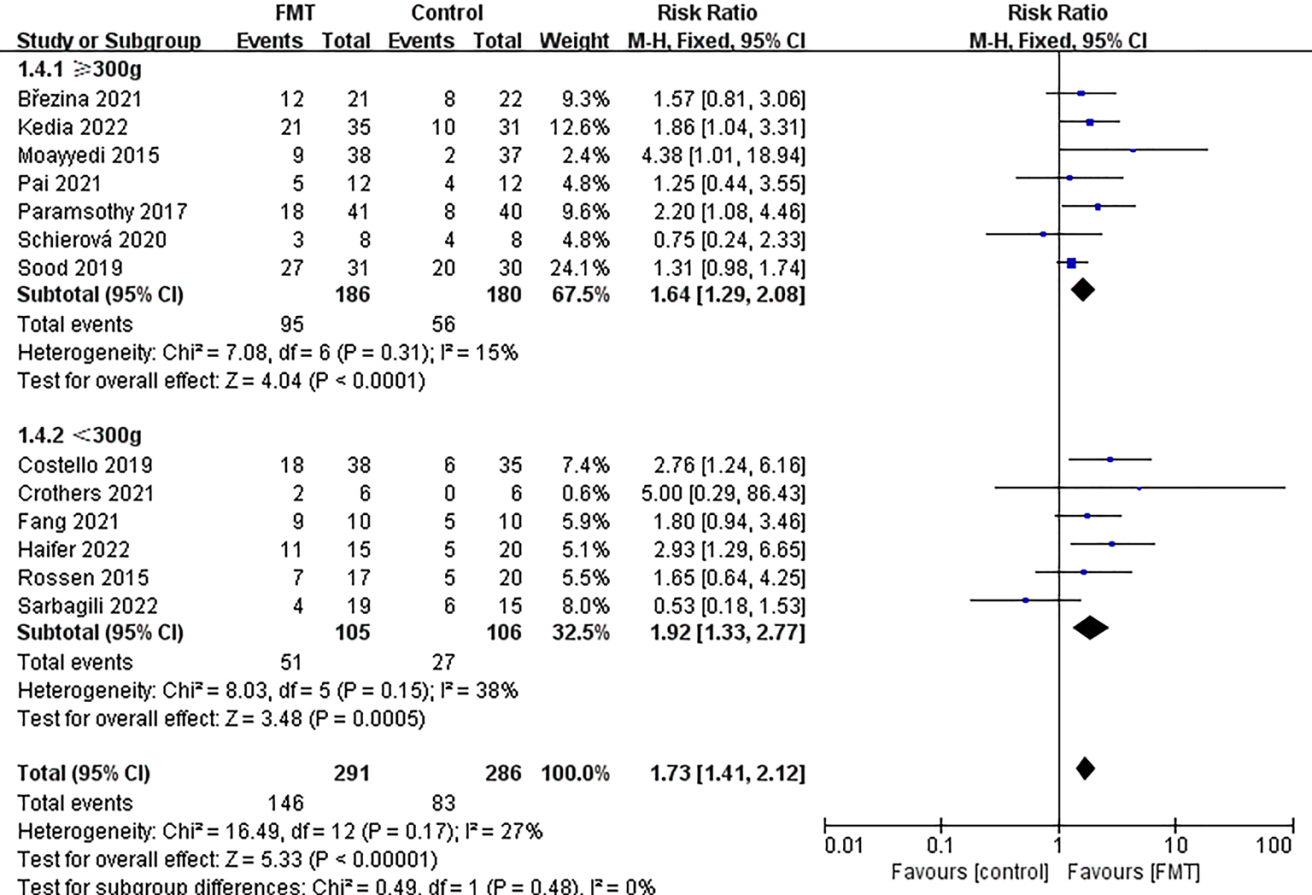
Subgroup analysis of clinical remission of total dose of FMT.

The studies were divided into six groups based on different factors. The first group was determined by the delivery route of the capsules, with one group receiving oral capsules and the other group receiving non-oral capsules (Fig. 5). The second group was divided based on donor type, with one group receiving FMT from a single donor and the other group receiving FMT from multiple donors (Fig. 6). The third group was determined based on the evaluation time since FMT, with one group being evaluated within 8 weeks and the other group being evaluated after 8 weeks (Supplementary Fig. 2). The fourth group was based on the total dose of FMT, with one group receiving a total dose of ≥ 300g and the other group receiving a total dose of < 300g (Fig. 7). The fifth group was categorized based on control mode, with one group given a placebo as the control and the other group given a non-placebo as the control (Supplementary Fig. 3). Finally, the sixth group was identified based on the publication time of literature, one before 2018 and the other after 2018 (Supplementary Fig. 4). The study results demonstrated the effectiveness of all subgroups in treating UC.

### Meta-analysis of endoscopic remission

**Figure 8:**
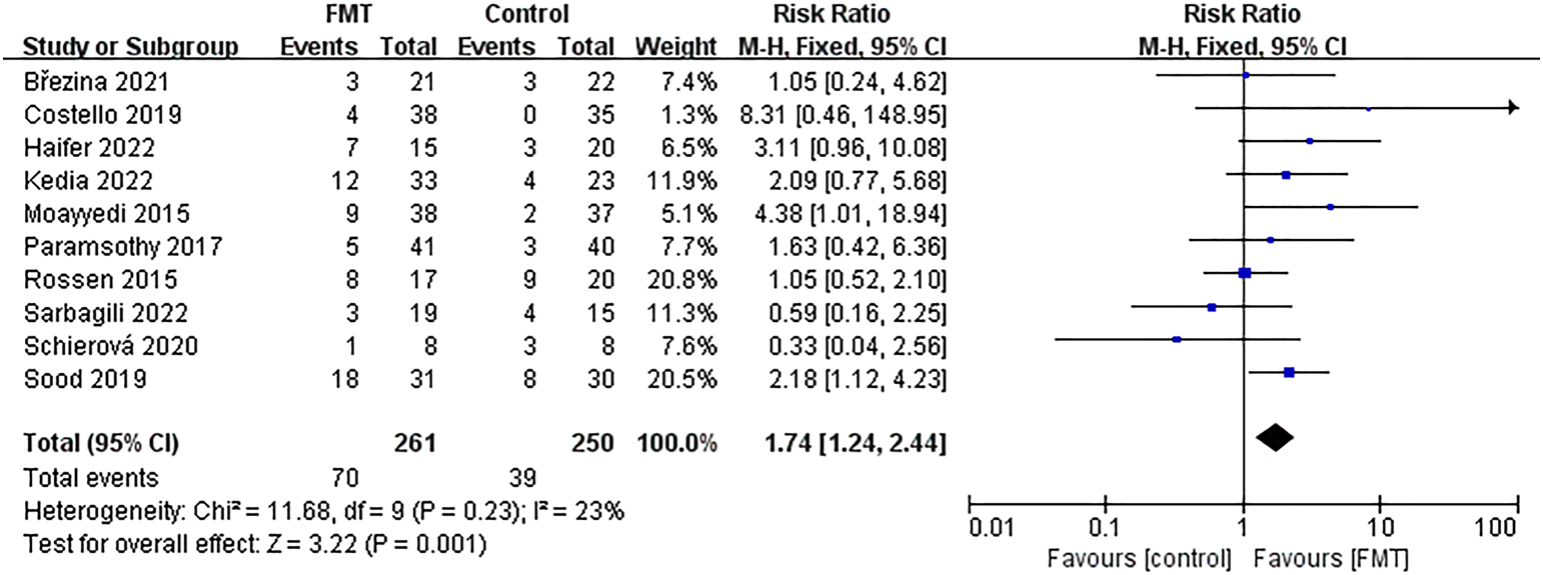
Forest plot for meta-analysis of endoscopic remission.

Ten studies were examined and reported endoscopic remission outcome data. Out of 261 patients in the FMT group, 70 achieved endoscopic remission (26.82%). In comparison, 39 out of 250 patients in the control group achieved remission (15.60%). The difference between the two groups was significant [*RR* = 1.74; 95% *CI* = (1.24, 2.44); *P* = 0.001]. The studies showed low heterogeneity, as confirmed through a meta-analysis (*Chi*^*2*^ = 11.68; *P* = 0.23; *I*^*2*^ = 23%) (Fig. 8).

We utilized a funnel plot to assess the presence of publication bias in the studies included (Supplementary Fig. 5). It was found that the distribution of results was symmetrical, which suggests that there is no bias present. Additionally, both Begg's and Egger's tests generated *P*-values of 0.8580 and 0.9578, respectively, which further support our conclusion that publication bias was not detected in this analysis.

### Subgroup analysis of endoscopic remission

**Figure 9:**
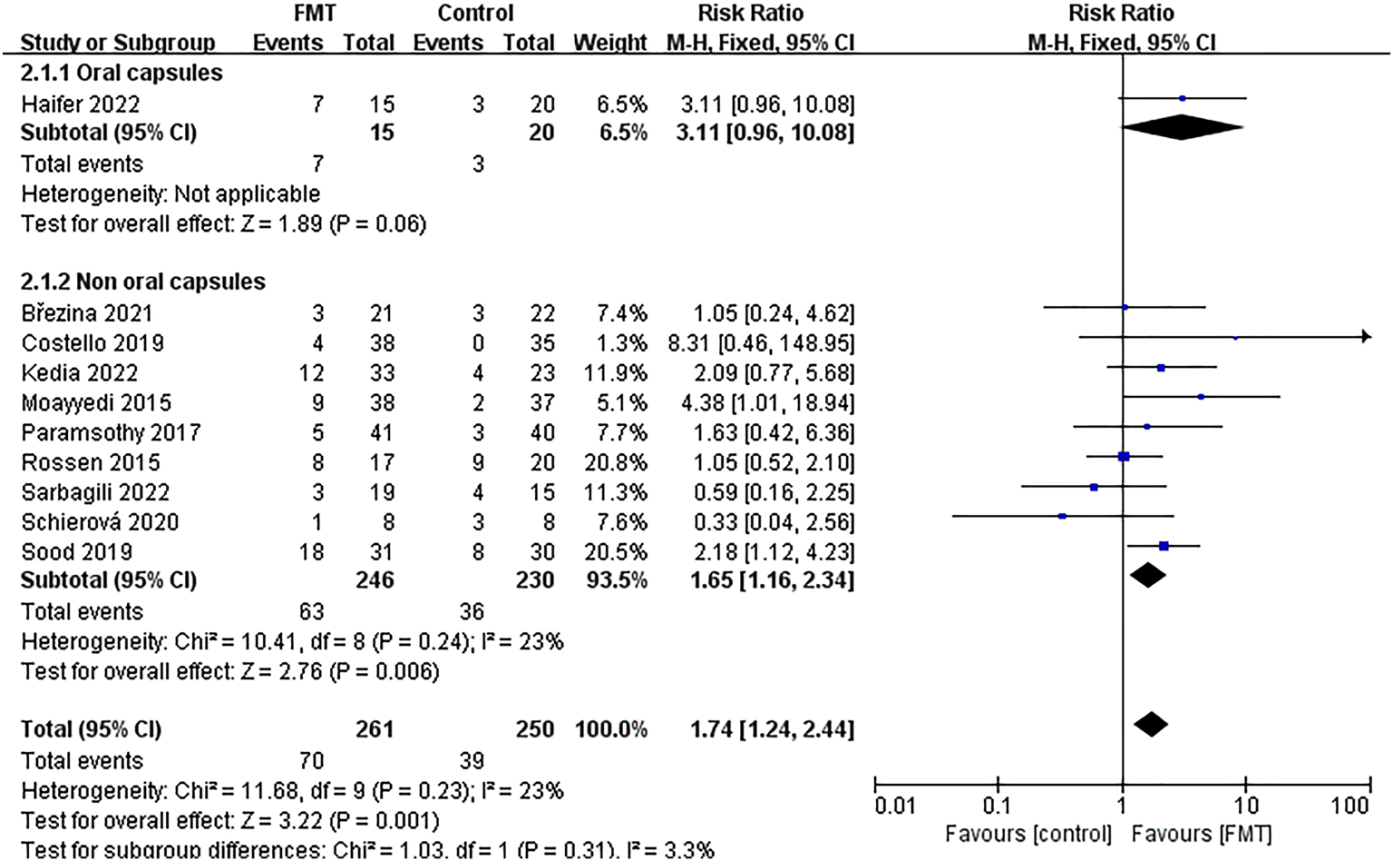
Subgroup analysis of endoscopic remission of delivery route.

**Figure 10:**
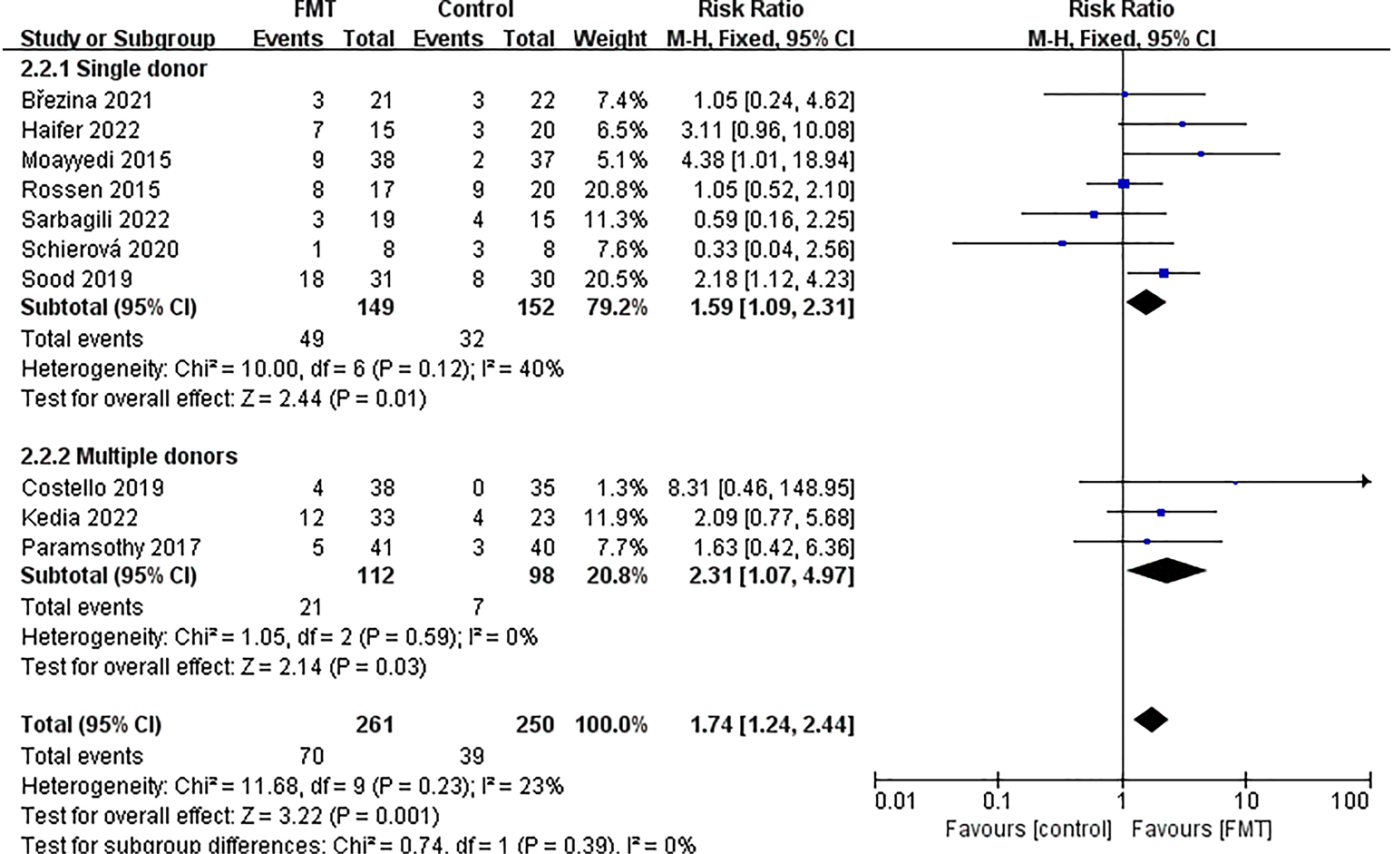
Subgroup analysis of endoscopic remission of donor type.

**Figure 11:**
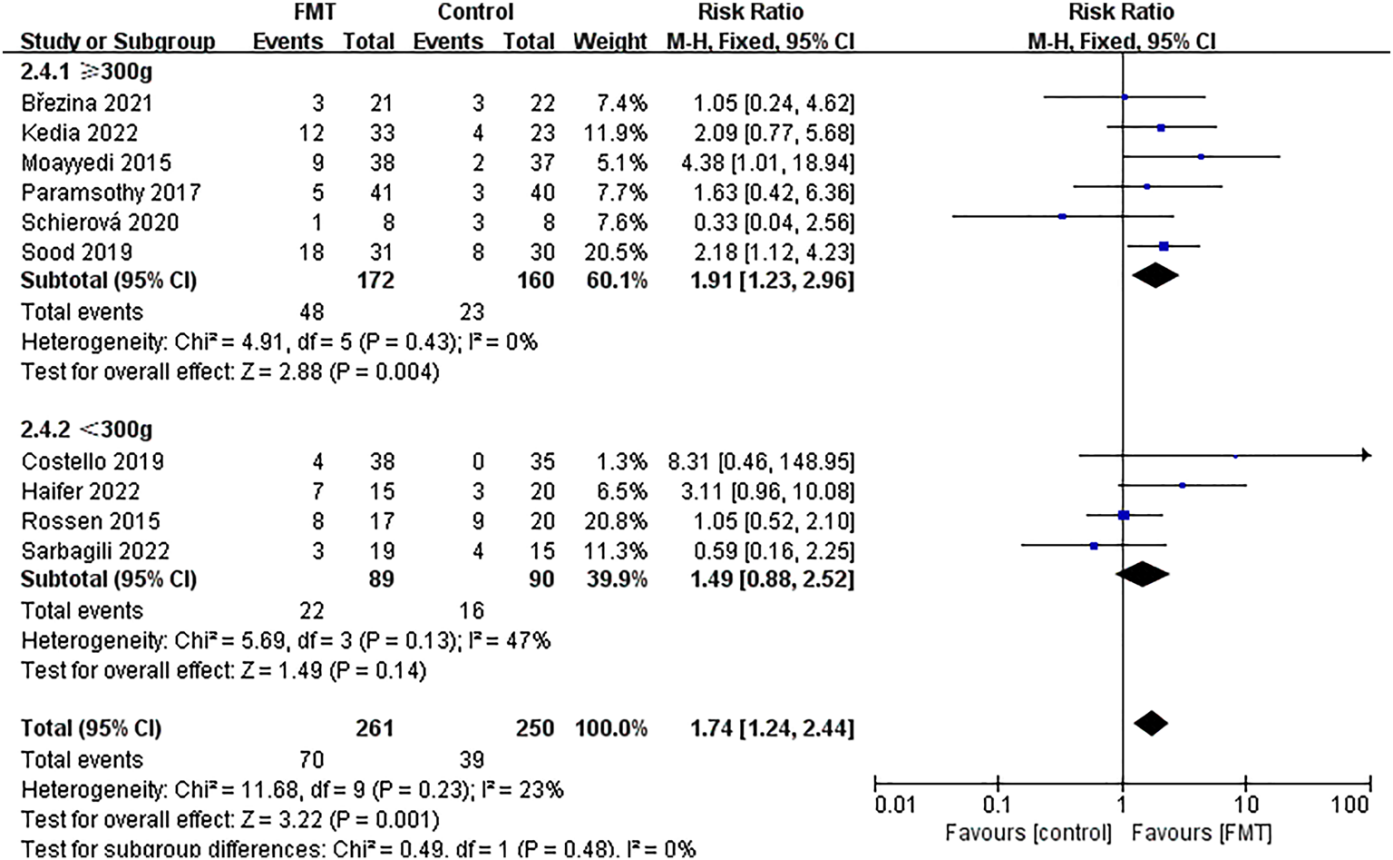
Subgroup analysis of endoscopic remission of total dose of FMT.

To further analyze subgroups based on delivery route, donor type, evaluation time, total dose of FMT, control mode, and the time of literature publication, we utilized endoscopic remission data. Subgroup analysis based on delivery route (Fig. 9) showed that the group that received non-oral capsules showed positive results in treating UC [63/246; 25.61%; *RR* = 1.65; 95% *CI* = (1.16, 2.34); *P* = 0.006], while there was no significant difference found in the treatment effect between the group receiving oral capsules and the control group [7/15; 46.67%; *RR* = 3.11; 95% *CI* = (0.96, 10.08); *P* = 0.06]. Subgroup analysis based on donor type (Fig. 10) demonstrated that both donor types were effective in the treatment of UC. Upon analyzing subgroups based on evaluation time (Supplementary Fig. 6), it was found that the group treated for 8 weeks yielded positive outcomes in the treatment of UC [40/184; 21.74%; *RR* = 2.23; 95% *CI* = (1.33, 3.75); *P* = 0.003], while there was no significant difference in the treatment effect between the group receiving treatment for more than 8 weeks and the control group [30/77; 38.96%; *RR* = 1.36; 95% *CI* = (0.88, 2.11); *P* = 0.17]. Subgroup analysis based on total dose of FMT (Fig. 11) showed that the group receiving ≥ 300 g showed a positive response in treating UC [48/172; 27.91%; *RR* = 1.91; 95% *CI* = (1.23, 2.96); *P* = 0.004], while there was no significant difference in the treatment effect between the group receiving < 300 g and the control group [22/89; 24.72%; *RR* = 1.49; 95% *CI* = (0.88, 2.52); *P* = 0.14]. Subgroup analysis based on control mode (Supplementary Fig. 7) suggested that the use of a placebo as a control group has been shown to have a positive effect in the treatment of UC [39/125; 31.20%; *RR* = 2.51; 95% *CI* = (1.51, 4.15); *P* = 0.0004], while the implementation of a non-placebo did not yield any discernible therapeutic effects in comparison to the control group [31/136; 22.79%; *RR* = 1.24; 95% *CI* = (0.78, 1.97); *P* = 0.37]. Following a subgroup analysis based on literature publication time (Supplementary Fig. 8), it was found that the group of studies published after 2018 demonstrated a positive impact on the treatment of UC [48/165; 29.09%; *RR* = 1.77; 95% *CI* = (1.17, 2.68); *P* = 0.007], while our findings revealed that the treatment effect in the group prior to the year 2018 was not significantly different from that of the control group [22/96; 22.92%; *RR* = 1.68; 95% *CI* = (0.94, 3.01); *P* = 0.08].

### Meta-analysis of adverse reactions

**Figure 12:**
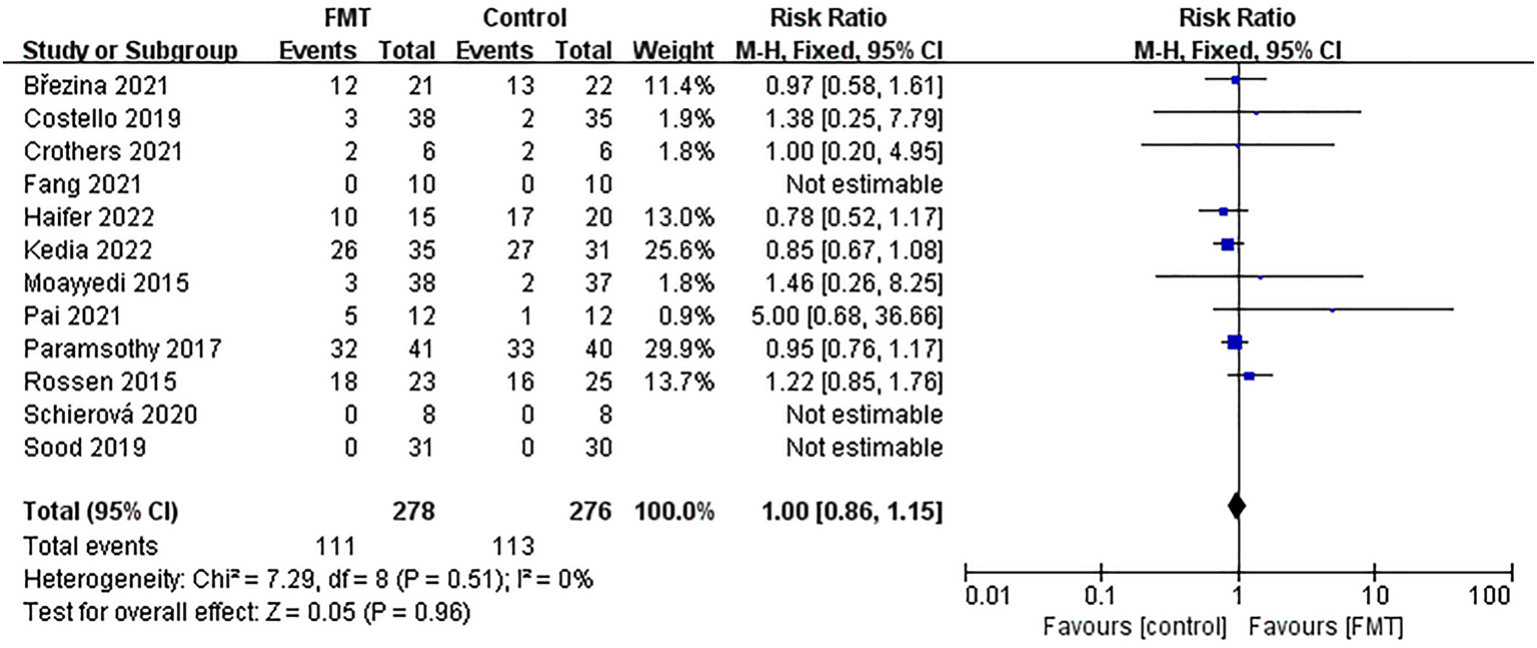
Forest plot for meta-analysis of adverse reactions.

In conducting our meta-analysis, we found that the RCT reported by Sarbagili et al. failed to provide a clear number of patients who experienced adverse reactions, which made it difficult to reconcile with data from other sources. Therefore, we only included data from the remaining 12 studies that explicitly reported the number of patients with adverse reactions when analyzing outcomes related to adverse reactions. Out of the 278 patients in the FMT group, 111 (39.93%) experienced adverse reactions, while 113 out of 276 (40.94%) patients in the control group had adverse reactions. However, the results were not statistically significant [*RR* = 1.00; 95% *CI* = (0.86, 1.15); *P* = 0.96], indicating no significant difference in the incidence of adverse reactions between the FMT group and control group. Additionally, meta-analysis revealed low heterogeneity between studies (*Chi*^*2*^ = 7.29; *P* = 0.51; *I*^*2*^ = 0%) (Fig. 12). Next, we employed a funnel plot to examine any publication bias in the included studies (Supplementary Fig. 9). Our analysis results in a symmetrical distribution. Other statistical tests, including Begg's test (*P* = 0.9453) and Egger's test (*P* = 0.2856) showed no evidence of publication bias. Furthermore, we conducted a comprehensive review of the available literature and meticulously differentiated adverse reactions reported in each study. Notably, the investigations conducted by Costello et al., Kedia et al., Haifer et al., Crothers et al., Brezina et al., and Pai et al. revealed a worsening of disease in patients following FMT, along with an aggravation of symptoms in the corresponding control groups. Additionally, other adverse events such as infection, temporary diarrhea, and abdominal distension were observed (Supplementary Table 1).

## Discussion

The prevalence of UC is on the rise worldwide, particularly in newly industrialized nations, which presents continuous obstacles for healthcare systems^21^. The treatment goal for UC is to achieve a rapid resolution of symptoms, promote mucosal healing, and improve patients' overall quality of life^22^. In order to thoroughly examine the effectiveness and safety of FMT for treating UC, we conducted a search and compiled 13 RCTs that were of high quality and focused on the use of FMT for UC treatment. There was a total of 580 participants with UC included in the study. As a result of our meta-analysis, we found that the FMT group demonstrated significantly improved rates of both clinical remission and endoscopic remission when compared to the control group. These findings were statistically significant and the level of heterogeneity among the studies was low. These findings indicate that the FMT group exhibited superior rates of clinical and endoscopic remission relative to the control group, suggesting that FMT has shown potential as a therapeutic intervention for inducing clinical remission in ulcerative colitis UC; however, achieving endoscopic remission and sustaining long-term remission still pose challenges.

In China, the average cost of treating a patient with inflammatory bowel disease between 2018 and 2019 was approximately US $11,668.68 ± 7944.44 for direct costs and US $74.90 ± 253.60 for indirect costs^23^. A research study conducted from 2016 to 2018 revealed that the total annual cost of treating UC patients in the United States was $36,441. Out of this total cost, $14,355 is attributed to medical expenses while drug costs make up the remaining $22,086^24^. UC patients often face significant financial burdens during treatment. However, studies show that FMT may be a cost-effective and beneficial alternative. In fact, research has found that switching from prior drug therapy to FMT can not only improve patients' quality of life but also reduce their financial burden, suggesting that FMT may be a promising treatment option for UC patients looking to alleviate financial strain^25^. FMT has proven to be a cost-effective approach to enhancing quality of life while also reducing both healthcare and social costs.

FMT has the potential to rectify dysregulated intestinal microbiota by increasing the proportion of beneficial bacteria, as well as the abundance and diversity of intestinal symbiotic microorganisms. These microorganisms are capable of releasing a variety of bioactive substances, including short-chain fatty acids (SCFAs), which possess immunomodulatory properties^26–28^.

Patients with UC exhibit a reduction in the levels of two critically important butyrate-producing bacterial species within *Firmicutes*, namely *Roseburia hominis* and *Faecalibacterium prausnitzii*, within their intestinal microbiota^29^. Butyrate, a short-chain fatty acid, is a primary energy source for colon epithelial cells^30^, and it also helps to maintain the epithelial barrier by decreasing intestinal permeability. This, in turn, protects the intestinal wall and reduces inflammation in the gut. FMT has been found to have the ability to reduce intestinal permeability by promoting the production of short-chain fatty acids, with a particular emphasis on butyrate. This can help to decrease the severity of UC^4^. Additionally, FMT has the capability to adjust the abundance of *Firmicutes*, *Bacteroidetes*, and *Proteobacteria*, ultimately leading to the gradual restoration of the intestinal microbiota to a normal state^31^. The potential health benefits of using FMT as a treatment for patients with UC are quite promising. A recent study has demonstrated that the combination of FMT and standard therapy proves to be more effective in achieving higher quality-adjusted life years (QALYs) than standard therapy alone, particularly for those with mild to moderate active UC^32^.

In our subgroup analyses that utilized endoscopic remission data, it was found that the oral capsules group, the group where evaluation time exceeded 8 weeks, the group that received a total dose of FMT < 300 g, the group that used a non-placebo control, and the group whose literature publication time was before 2018 did not show significant differences in treatment effects when compared to the control group. The rest of the subgroups showed promising treatment effects for UC. However, our research indicates that administering FMT through oral capsules yields superior clinical remission outcomes compared to other delivery methods. Multiple studies have demonstrated that oral capsules are no less effective than colonoscopy and are a safe and well-tolerated option for treating UC patients^33, 34^. Oral FMT capsules present a convenient alternative to the burdensome colonoscopy or enema method for treatment delivery. Patients can take the capsules over an extended period of time and can receive them either in an outpatient setting or the comfort of their own homes. Additionally, patients who cannot tolerate the colonoscopy method can still receive FMT through this oral route^35^. The capsule route of FMT has emerged as a promising new approach that is both convenient and acceptable to patients. However, our subgroup analyses of clinical remission and endoscopic remission have yielded inconsistent results. We suspect that this is due to the limited number of RCTs associated with the existing “UC treatment with FMT oral capsules”. To achieve greater accuracy in our results, it is necessary to explore a broader range of relevant studies in future analyses.

The endoscopic remission effect in the group that underwent evaluation for more than 8 weeks was not significantly different from the control group. This indicates the need for further investigation into the effectiveness of FMT as a long-term treatment for UC. Besides, the total dose of FMT < 300 g group exhibited no significant difference in endoscopic remission effect compared to the control group. These findings suggest that the amount of FMT administered correlates with its efficacy in inducing endoscopic remission in UC. Moreover, the efficacy of FMT in treating UC has steadily increased over the past five years, particularly in the group of literature published after 2018. This group showed significant improvements in endoscopic remission compared to the control group, which reflects that FMT has become a more professional and mature therapy for UC in recent years. Although the subgroup analysis of clinical and endoscopic remission yielded slightly different results, it is evident that FMT is a successful treatment for UC.

Although the effectiveness of FMT has been demonstrated, its safety remains a critical consideration. In a review of 129 studies that evaluated FMT as a treatment for various diseases, 19% reported adverse events related to FMT, with 1.4% reporting serious adverse events^36^. However, after conducting a meta-analysis of adverse reactions, we found no significant differences in the incidence of adverse reactions between the group receiving FMT and the control group. In the course of studying UC treatments, it has been observed that patients may experience mild adverse reactions such as abdominal pain, distention, diarrhea, nausea, and fever. Although these reactions were generally self-limiting, it is still important to closely monitor the safety of FMT treatments to ensure optimal patient outcomes. To maximize the safety of FMT, several important aspects must be considered. These include strict screening and management of the donor to prevent any transmission of infectious pathogens from the feces to the recipient^37^. It is also crucial to ensure the quality and safety of the FMT product during preparation, select the appropriate transplantation period based on the patient's condition, and carefully match the donor with the recipient. These methods are all essential for improving the success rate of FMT and mitigating any associated risks.

In this study, compared with the previous meta-analysis, our meta-analysis added the most recent studies with updated data on the efficacy and safety of FMT. However, the studies we included still had some limitations, such as the evaluation time was not completely consistent and the distinction of patient types was not clear across studies. These limitations will be improved in the next studies.

## Conclusions

FMT is a promising treatment for UC, with demonstrated clinical and endoscopic remission rates. However, the concerns around its safety during treatment require additional attention and improved measures to ensure its safety. By focusing on improving safety measures and success rates, we can gain a better understanding of how effective and safe this approach truly is.

### Supplementary Information

Supplementary Information 1.

Supplementary Information 2.

Supplementary Information 3.

Supplementary Information 4.

Supplementary Information 5.

Supplementary Information 6.

Supplementary Information 7.

Supplementary Information 8.

Supplementary Information 9.

Supplementary Information 10.

Supplementary Information 11.

## Data Availability

The data underlying this article are available in the article and its supplementary material.
