# Supplementary figures and images for: Efficacy and safety of fecal microbiota transplantation in the treatment of ulcerative colitis: a systematic review and meta-analysis
Source: Sci Rep. 2023 Sep 3;13:14494. doi: 10.1038/s41598-023-41182-6 (PMC10475461; doi:10.1038/s41598-023-41182-6)

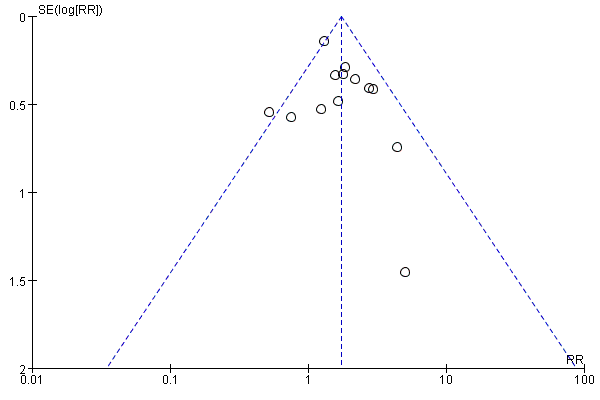

Supplement: Supplementary file 1 — Supplementary Information 1. [file 41598_2023_41182_MOESM1_ESM.png]

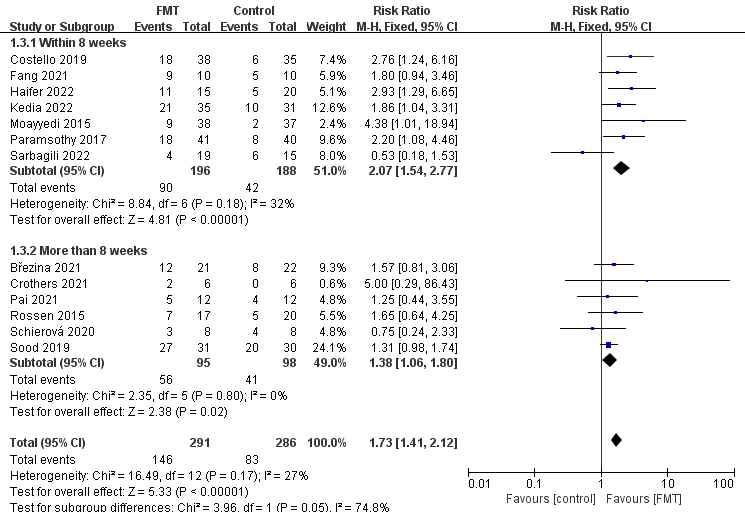

Supplement: Supplementary file 2 — Supplementary Information 2. [file 41598_2023_41182_MOESM2_ESM.png]

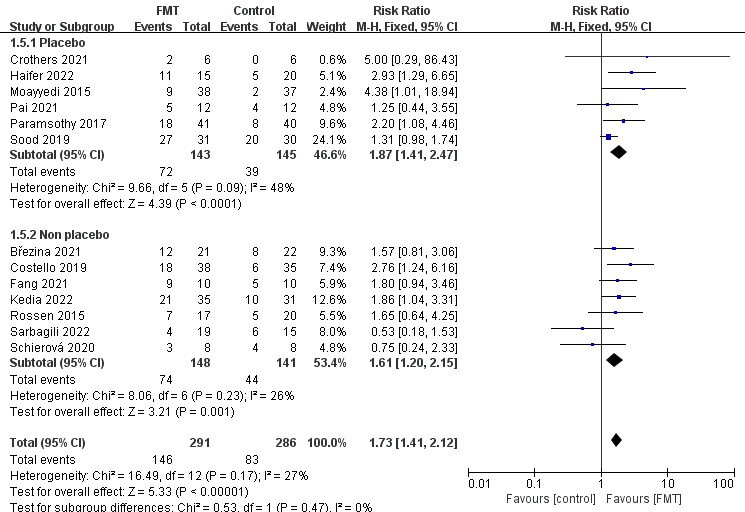

Supplement: Supplementary file 3 — Supplementary Information 3. [file 41598_2023_41182_MOESM3_ESM.png]

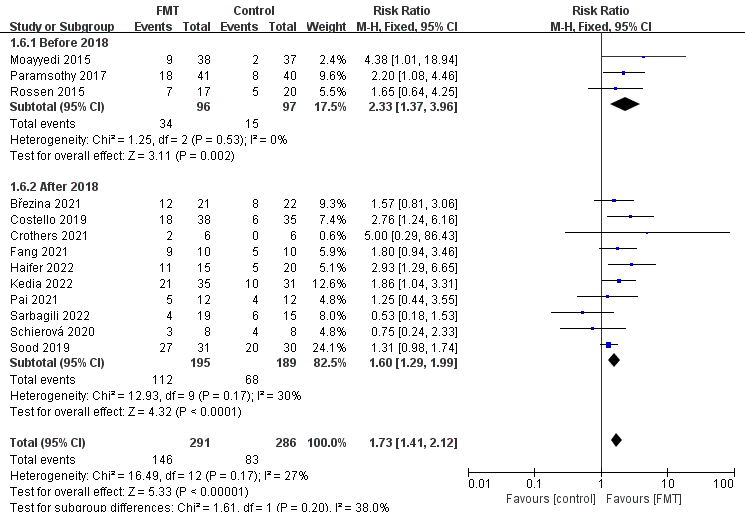

Supplement: Supplementary file 4 — Supplementary Information 4. [file 41598_2023_41182_MOESM4_ESM.png]

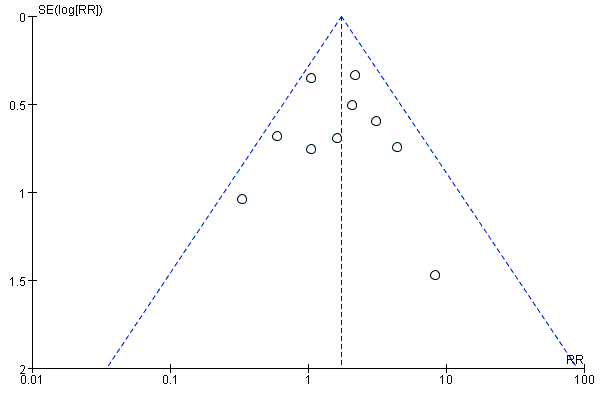

Supplement: Supplementary file 5 — Supplementary Information 5. [file 41598_2023_41182_MOESM5_ESM.png]

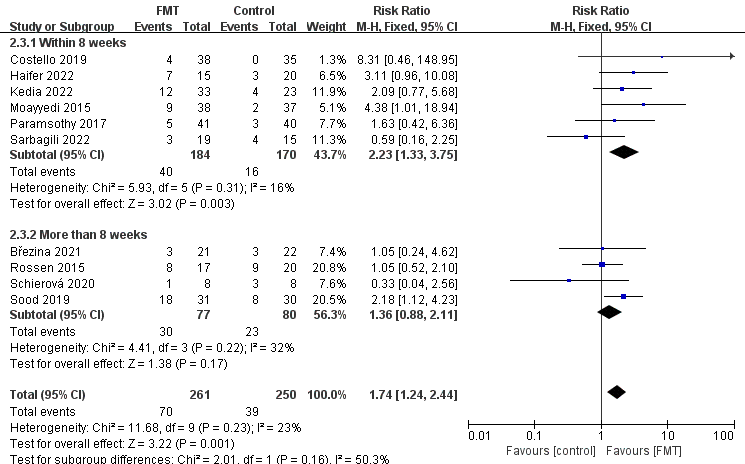

Supplement: Supplementary file 6 — Supplementary Information 6. [file 41598_2023_41182_MOESM6_ESM.png]

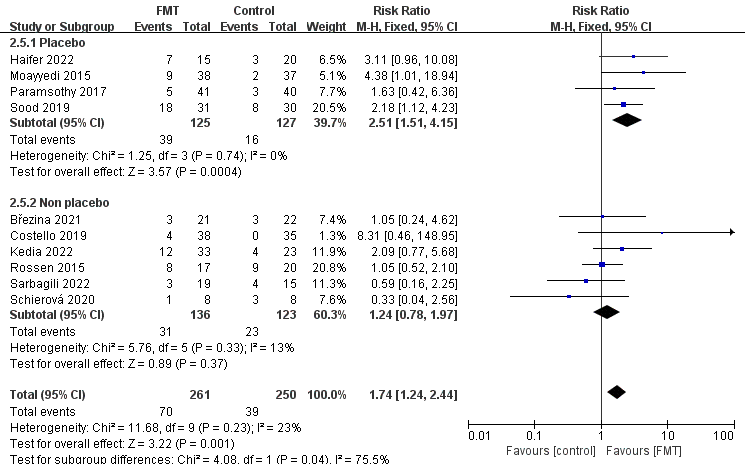

Supplement: Supplementary file 7 — Supplementary Information 7. [file 41598_2023_41182_MOESM7_ESM.png]

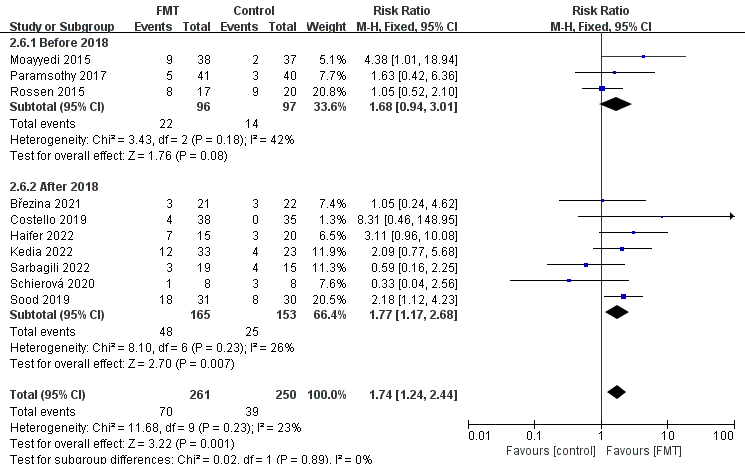

Supplement: Supplementary file 8 — Supplementary Information 8. [file 41598_2023_41182_MOESM8_ESM.png]

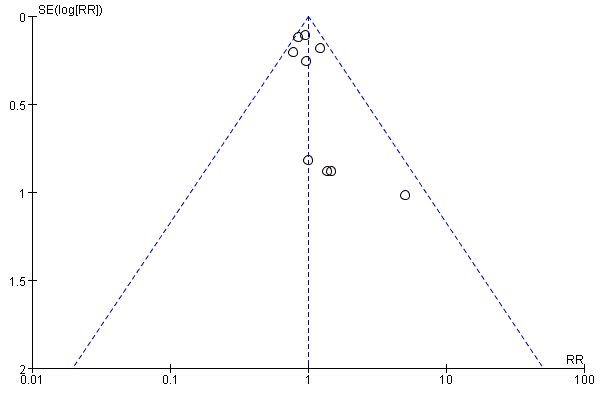

Supplement: Supplementary file 9 — Supplementary Information 9. [file 41598_2023_41182_MOESM9_ESM.png]
