# Supplementary material for: Efficacy and safety of fecal microbiota transplantation in the treatment of ulcerative colitis: a systematic review and meta-analysis
Source: Sci Rep. 2023 Sep 3;13:14494. doi: 10.1038/s41598-023-41182-6 (PMC10475461; doi:10.1038/s41598-023-41182-6)
Supplement: Supplementary file 10 — Supplementary Information 10. [file 41598_2023_41182_MOESM10_ESM.docx]

**Supplementary figure legends**

Supplementary Figure 1 Funnel plot of publication bias in clinical remission.

Supplementary Figure 2 Subgroup analysis of clinical remission of evaluation time.

Supplementary Figure 3 Subgroup analysis of clinical remission of control mode.

Supplementary Figure 4 Subgroup analysis of clinical remission of literature publication time.

Supplementary Figure 5 Funnel plot of publication bias in endoscopic remission.

Supplementary Figure 6 Subgroup analysis of endoscopic remission of evaluation time.

Supplementary Figure 7 Subgroup analysis of endoscopic remission of control mode.

Supplementary Figure 8 Subgroup analysis of endoscopic remission of literature publication time.

Supplementary Figure 9 Funnel plot of publication bias in adverse reactions.

**Supplementary Table**

Supplementary Table 1 The adverse events in each of the studies.
